# Effectiveness of remineralising agents in prevention and treatment of orthodontically induced white spot lesions: a protocol for a systematic review incorporating network meta-analysis

**DOI:** 10.1186/s13643-019-1253-8

**Published:** 2019-12-29

**Authors:** Huimin Hu, Chong Feng, Zhaowei Jiang, Lufei Wang, Sonu Shrestha, Xiaoming Su, Yu Shu, Long Ge, Wenli Lai, Fang Hua, Hu Long

**Affiliations:** 1 Department of Orthodontics, State Key Laboratory of Oral Diseases, National Clinical Research Center for Oral Diseases, West China Hospital of Stomatology, No. 14, Section 3, Ren Min South Road, Chengdu, 610041 China; 2 School of Medicine, University of Nankai, 94 Weijin Road, Tianjin, 300071 Nankai China; 3 Department of Oral and Craniofacial Health Sciences, University of North Carolina Adams School of Dentistry, 385 South Columbia Street, Chapel Hill, NC 27599 USA; 4 Department of Medical Informatics, Biometry, and Epidemiology (IBE), Ludwig-Maximilians University of Munich, Marchioninistrasse 15, 81377 Munich, Germany; 5 Sichuan University Library, 29 Wangjian Road, Chengdu, Sichuan China; 6 Evidence-based Social Science Research Center & Department of Social Medicine and Health Management, School of Public Health, Lanzhou University, 222 Tianshui South Road, Lanzhou, Gansu China; 7 Department of Orthodontics & Centre for Evidence-Based Stomatology, Hubei-MOST KLOS & KLOBM, School & Hospital of Stomatology, Wuhan University, Wuhan, 430072 Hubei China; 8 Division of Dentistry, School of Medical Sciences, Faculty of Biology, Medicine and Health, University of Manchester, Manchester Academic Health Science Centre, Nell Lane, Manchester, M20 2LR UK

**Keywords:** Orthodontic, White spot lesion, Tooth remineralisation, Network meta-analysis

## Abstract

**Background:**

White spot lesions (WSLs) are common adverse effects in fixed orthodontic treatment. Remineralising agents are widely used to prevent WSLs formation and are the first-line treatment for existing WSLs. Previous systematic reviews have evaluated the effectiveness of remineralisation agents in the management of WSLs. However, their conclusions were contradictory. The objective of this study will be to compare the effectiveness and safety of current remineralising agents used in the management of WSLs in patients treated with fixed orthodontic appliances in any orthodontic setting.

**Methods:**

Literature searches will be conducted in several electronic databases (from inception onwards): MEDLINE (via Ovid), Scopus, Embase, the Web of Science, and the Cochrane Central Register of Controlled Trials (CENTRAL), among others. Grey literature will be identified through searching clinical trials registries. Randomised controlled trials that compared the effectiveness of different remineralisation agents in the prevention and treatment of WSLs will be included. Two researchers will independently screen all citations, full-text articles, and abstract data. The study risk bias will be appraised using an appropriate tool. The primary outcomes will be WSLs incidence and severity of WSLs. Secondary outcomes will be subjective measures of WSLs and adverse effects. The mean difference (MD) and relative risk (RR) with corresponding 95% confidence intervals (CI) will be chosen as effect measures for continuous and binary outcomes, respectively. If feasible, fixed and random-effects pairwise meta-analyses and frequentist network meta-analyses will be conducted where appropriate.

**Discussion:**

This network meta-analysis will compare the effectiveness of remineralising agents in the prevention and treatment of orthodontically induced WSLs. By integrating the evidence from direct and indirect comparisons and ranking all evaluated interventions, our findings have the potential to help clinicians make more accurate treatment decisions.

**Systematic review registration:**

PROSPERO CRD42019116852, registered on March 15, 2019

## Background

### Description of the condition

White spot lesions (WSLs), defined as ‘subsurface enamel porosity from carious demineralisation’ that manifest as ‘milky white opacities located around the bracketed gingival area in the tooth surface’, are one of the most common adverse effects in fixed orthodontic treatment [1–3].

Demineralisation occurs when a low pH persists for a longer period of time and large amounts of calcium ions are released from the enamel of the tooth (mainly through hydroxyapatite; HAP) [4]. In addition, bonding attachments hinder conventional oral hygiene and limit natural oral self-cleaning mechanisms, which leads to accumulation of plaque and a lower pH [5, 6]. WSLs cause cosmetic problems in anterior teeth and influence patients’ satisfaction with their smile [7–9]. The reported prevalence of WSLs was relatively high, ranging from 23.4 to 49.6% after debonding, depending on the measurement method or criteria [7, 10]. Along with the importance of the prevention and treatment of WSLs with remineralising agents, the safety of the remineralising agents is equally important, since an intake of excess fluoride may lead to fluoride toxicity. As such, proper techniques and safety measures must be adapted when applying remineralising agents.

### Description of the intervention

Remineralising agents are the most commonly used interventions in the prevention of WSLs and the first-line treatment for post-orthodontic WSLs. Topical fluoride use is the most reported method of prevention and treatment for early enamel demineralisation, including fluoride materials of different concentrations (in the form of bonding materials, sealants, gels, mouth rinses, toothpastes, or varnishes) [11–13]. There are alternatives to these, such as casein phosphopeptide-amorphous calcium phosphate (CPP-ACP) creams, casein phosphopeptide-amorphous calcium phosphate with fluoride (CPP-ACFP), and bioactive glass toothpaste, among others [14–16].

Many interventions are self-applied agents, such as mouth rinses and toothpastes containing fluoride. Some interventions are performed by dentists, such as a fluoride varnish (Duraphat; Colgate-Palmolive, New York, NY) or a fluoride film (Sheer; CAO Group, West Jordan, UT) with minimal clinical chair time [15, 17]. Almost all of these interventions are combined with tooth-brushing guidance and oral hygiene education [14, 15].

### How the intervention might work

Almost all preventive measures and remineralising interventions aim to enhance enamel resistance to acid and prompt the process of remineralisation to reverse the caries process. Prior studies have shown that during the remineralisation process, enamel lesions preferentially adsorb fluoride ions onto partial enamel lesions with demineralised HAP crystals or redeposit fluorohydroxyapatite (FHAP) [17]. Therefore, increased fluoride can enhance remineralisation and form a low-solubility veneer, which is an acid-resistant mineral on the remineralising crystals [18].

CPP-ACP has a beneficial sub-surface effect, whereby its milk-protein-based formulation can promote the natural salivary healing process. In addition, its nanoclusters of ACP are small enough to access demineralised areas through an existing remineralised surface zone [19–21].

Bioactive glass is a ceramic material consisting of amorphous sodium calcium-phosphosilicate [22]. It can release sodium ions for exchange with hydrogen cations to release calcium and phosphate ions, whereby the topical pH increase would precipitate the extra calcium and phosphate ions to form a calcium phosphate layer. This layer crystallises into hydroxycarbonate apatite (HCA) as these reactions continue [23]^.^

### Why it is important to do this review

Previous studies have compared the effectiveness of many remineralising agents in WSLs prevention and treatment [13–15]. However, many of these studies are not well-designed and have limited methodology. In addition, there are differences in purpose, design, and treatment among current studies regarding WSLs remineralisation agents [18, 24–26]. Furthermore, previous studies have not clearly explored the safety of remineralising agents.

Several systematic reviews have been published to investigate the comparative effectiveness of remineralising agents for WSLs prevention and treatment [27–29]. However, all these systematic reviews have only found pairwise evidence from head-to-head comparisons and have thus failed to evaluate the comparative effectiveness of all available remineralising agents. In addition, the effectiveness rankings of the remineralising agents are still unclear. Network meta-analysis (NMA) can integrate the evidence from direct and indirect comparisons. It enables inference about every possible comparison between a pair of interventions in the network, even when some comparisons have never been evaluated in a trial. Through the comparison of multiple interventions, we can rank all evaluated interventions with minimal adverse effects [30–32].

**Fig. 1:**
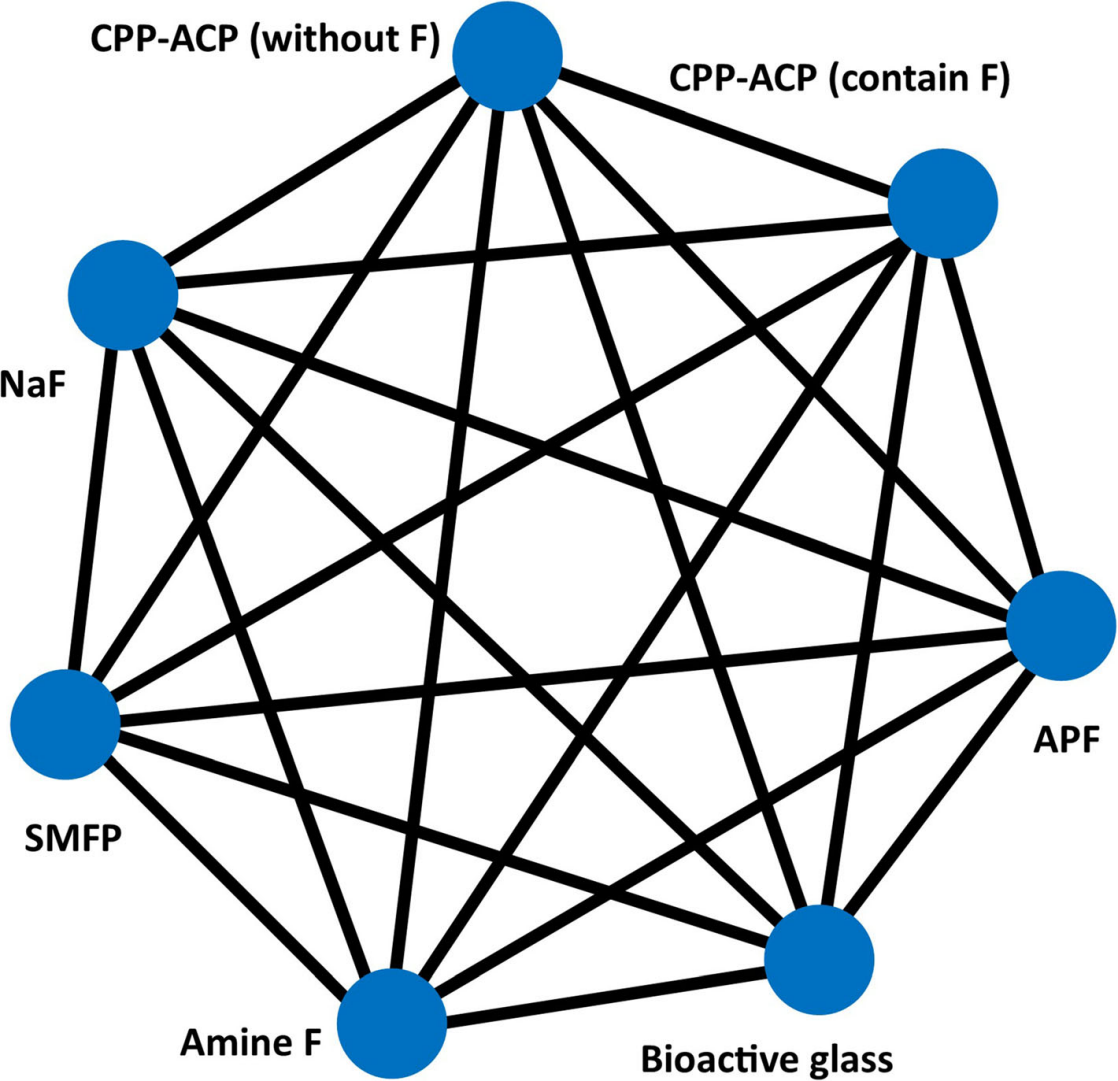
The network plot of all possible direct comparisons between the eligible interventions

Therefore, this study will involve a systematic review and frequentist framework network meta-analysis to compare different remineralising agents for the prevention and treatment of WSLs containing different concentrations of fluoride, CPP-ACP, CPP-ACFP, bioactive glass, and the safety of the current remineralising agents (Fig. 1).

### Objective

In this review, we will compare the effectiveness and safety of remineralising agents used in the prevention and treatment of WSLs in patients treated with fixed orthodontic appliances in any type of orthodontic setting.

## Methods

The review protocol was written in accordance with the Preferred Reporting Items for Systematic Reviews and Meta-Analysis Protocols (PRISMA-P) and the PRISMA checklist for Reporting Systematic Reviews incorporating Network Meta-analyses [33, 34]. The protocol was registered in PROSPERO (CRD42019116852). PRISMA-P is included in Additional file 1.

### Criteria for considering studies for this review

The eligibility criteria for our systematic review have been developed according to the PICOS acronym. These criteria are presented here and details are summarised in Table 1 [35].

1. Patients. Patients with at least one white spot lesion on the labial surface of the teeth induced by fixed orthodontic treatment or who will receive fixed orthodontic treatment and will be observed about WSLs with no restrictions on their sex, age, city, country, ethnicity, and socio-economic status.
2. Interventions. Remineralised agents used for already formed WSLs induced by orthodontic treatment or prevention of orthodontically induced WSLs formation. Different forms/active ingredients of remineralised materials will be distinguished as different interventions (e.g. NaF varnish and difluorosilane varnish will be distinguished as two interventions, NaF varnish, and NaF gel will be distinguished as two interventions). Similar forms/active ingredients of remineralised materials regardless of intervention doses, administration frequencies, and duration of the interventions will be merged into the same node, so there will not be too many disconnected nodes that make the NMA unable to conduct.
3. Comparators. Any other kind of remineralised agents or control/placebo.
4. Outcomes. WSLs incidences, lesion severity (measured by WSL index, enamel decalcification index, DIAGNOdent pen reading, quantitative light-induced fluorescence, etc), adverse effect event, other outcomes evaluate WSLs.
5. Study designs. Only RCTs (randomised clinical trials) will be included.

**Table 1:** Inclusion and exclusion criteria

Domain	Inclusion	Exclusion
Participants	• Patients with at least one white spot lesion on the labial surface of the teeth induced by fixed orthodontic treatment. • Patients who will receive fixed orthodontic treatment and will be observed about WSLs. • No restrictions on patients’ sex, age, city, country, ethnicity, and socio-economic status.	• Laboratory animal. • Patients with WSLs but were not induced by orthodontic treatment. • Patients with any illness potentially affecting the study outcome, such as enamel hypoplasia, craniofacial deformities, ongoing medication, and so on. • Patients with congenital anomalies for example with cleft lip and palate.
Interventions	• Remineralised agents used for already formed WSLs induced by orthodontic treatment or prevention of orthodontically induced WSLs formation. • Different forms/active ingredients of remineralised materials will be distinguished as different interventions(e.g. NaF varnish and difluorosilane varnish will be distinguished as two interventions, NaF varnish, and NaF gel will be distinguished as two interventions). • Similar forms/active ingredients of remineralised materials regardless of intervention doses, administration frequencies and duration of the interventions will be merged into the same node, so there will not be too many disconnected nodes that make the NMA unable to conduct.	• Non-remineralised methods for prevention and treatment orthodontically induced WSLs, such as bleaching, micro-abrasion, and resin infiltration. • If remineralised methods and non-remineralised methods were jointly used as an intervention in the same study, we will include the article but not pool the data.
Comparisons	• No treatment or placebo. • Any other kind of remineralised agents.	–
Outcome	• Lesion severity (measured by WSL index, enamel decalcification index, DIAGNOdent pen reading, quantitative light-induced fluorescence, etc.). • Lesion transition (progression, stability or regression). • WSLs prevalence (in the prevention of WSLs). • Other outcomes evaluate WSLs.	–
Study design	• Randomised controlled trials (parallel or clustered).	• Non-randomised prospective or retrospective studies. • Split-mouth trials, which are susceptible to “carry-across effect” and the resultant bias. • Case reports/ case series. • Non-clinical studies (in vitro, ex vivo, in silico, etc.). • Systematic review.
Timing	• Any time points.	–
Setting	• No restrictions by type of setting. e.g. university or private practice,.	–
Language	• Studies written in all languages.	–
Other imitations	• No other limitations will be imposed on unpublished studies, studies of all durations and those conducted during all points in time are eligible for inclusion.	–

No other limitations will be imposed on unpublished studies, the language of publication, study settings, studies of all durations, and those conducted during all points in time are eligible for inclusion.

We assume that patients who fulfil the inclusion criteria are equally eligible to be randomised to any of the interventions we plan to compare.

### Search methods for identification of studies

#### Electronic searches

The following electronic databases will be searched (from inception onwards): The Cochrane Central Register of Controlled Trials (CENTRAL), PubMed (Ovid), Embase, Scopus, Web of Science, Chinese Biomedical Literature Database, China National Knowledge Infrastructure, Wan Fang Database, VIP, and Google Scholar. We will use a combination of medical subject headings and free texts related to ‘orthodontic’, ‘randomized controlled trial’, and ‘white spot lesions’ for the literature search (a draft of the MEDLINE search strategy is included in Additional file 2). No restrictions were set on language or publication date. The search strategies will be peer-reviewed, according to the PRESS 2015 Guideline Statement for the PRESS Peer Review of Electronic Search Strategies (PRESS) [36, 37] (Additional file 3).

#### Searching other resources

- A manual search of the reference lists of studies identified through electronic searches, relevant systematic reviews and narrative reviews
- US National Institutes of Health Ongoing Trials Register ClinicalTrials.gov (http://clinicaltrials.gov/)
- World Health Organization International Clinical Trials Registry Platform (apps.who.int/trialsearch)

### Study records

#### Selection of studies

**Fig. 2:**
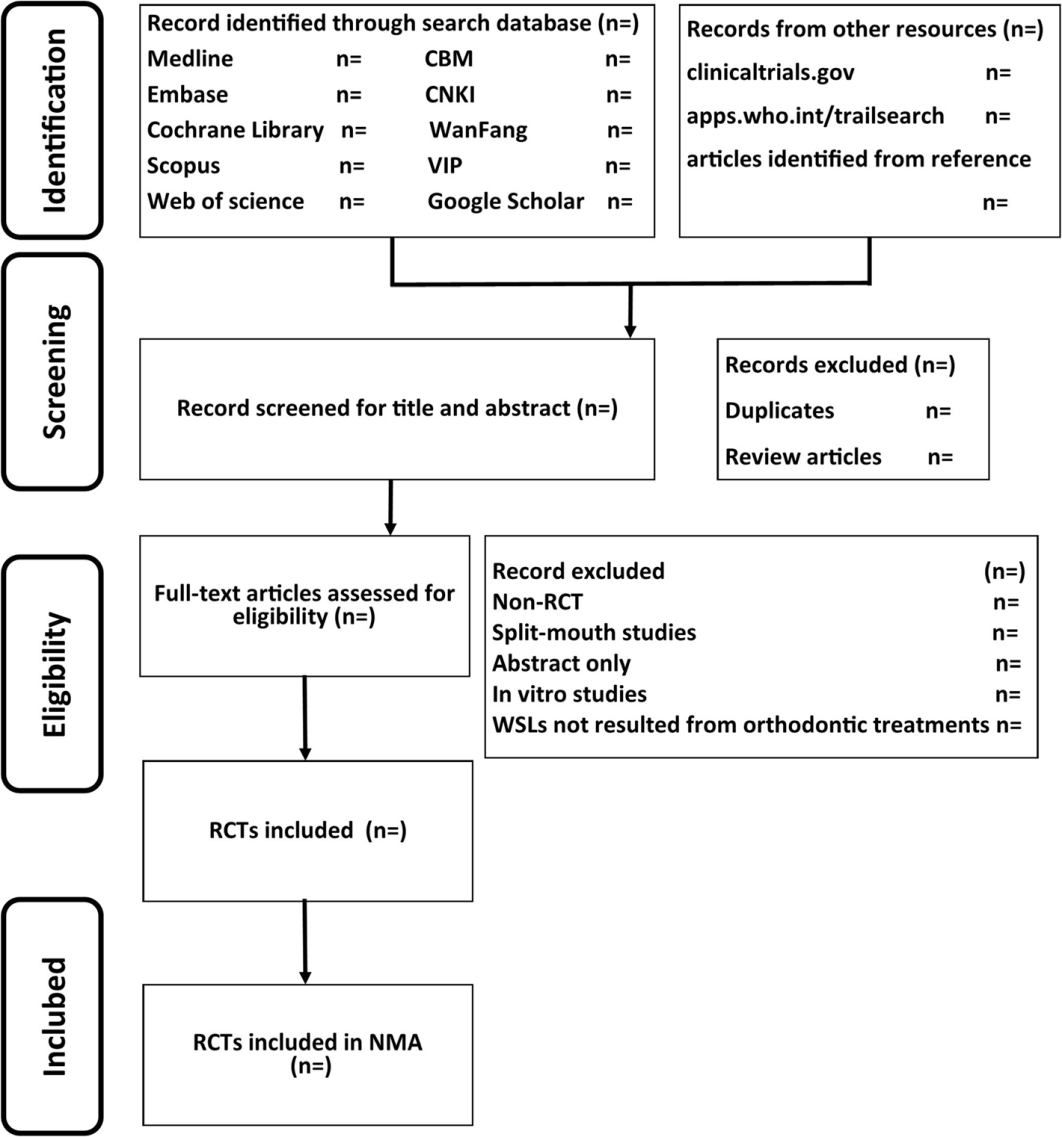
The PRISMA flow diagram for study screening

Two authors will independently perform, in duplicate, screening of titles and abstracts according to pre-determined eligibility criteria. Thereafter, for titles that may be eligible, their full texts will be examined. When necessary, we will seek more information from study authors to confirm eligibility for these studies. All disagreements will be resolved by discussion or by involving a third assessor. We will record the reasons for excluding trials in the ‘Characteristics of excluded studies’ table. The process will be presented in a PRISMA flow diagram for study screening (Fig. 2). All records identified in the databases will be collected in the reference management software EndNote® X8 (Thomson Reuters, New York, NY).

### Data extraction

Two reviewers will extract data from each included study using Microsoft Excel 2010 (Microsoft, Redmond, WA) with a specifically developed data extraction form, independently and in duplicate. Data extraction forms will be piloted initially on a small number of included studies. All disagreements will be resolved by discussion or by involving a third assessor. We will record sources of funding if stated. We will record the study details in the ‘Characteristics of included studies’ table (Additional file 4). We will collate multiple reports of the same study. We will extract estimates of 2 × 2 tables (dichotomous data), means and standard deviations from effect estimates, confidence intervals, and other forms of data. From each trial, the following data/information will be collected [35]:

- Study characteristics (author, year of publication, study design, number of arms, sample size, duration of follow-up, withdrawals), randomisation (individual or cluster)
- Participant characteristics (age, sex, number of participants)
- Intervention and comparator details (preventative or therapeutic methods, intervention performer, materials and techniques used, frequency or duration, active ingredients, concentration/dosage form, time of follow-up)
- Outcome (WSLs incidences, lesion severity, adverse effect event, other outcomes evaluate WSLs). Where possible, we will extract data at the arm level, not summary effects. If outcome results are not directly provided and it is feasible, we will do the data imputation.
- Notes: sponsorship/funding for the trial and notable conflicts of interest of trial authors

### Outcomes and prioritisation

The primary outcomes are the severity of WSLs at pre-intervention and at last available follow-up assessment measured by objective methods as follows:

- WSLs incidence [3]
- The severity of WSLs: DIAGNOdent pen reading (KaVo Dental, Biberach an der Riss, Germany) [38], QLF (quantitative light-induced fluorescence parameters) [39]

Secondary outcomes will be subjective measures of WSLs as follows:

- Subjective measures: WSL index (such as Gorelick method [7], International Caries Detection and Assessment System (ICDAS) [40], ICDAS-II [41]), enamel decalcification index [42]
- Adverse effects (e.g. yellowing of teeth, gastrointestinal effects, or nausea)

Most outcomes are observed among multiple time points because remineralising agents require long-term use. While combining outcomes that were measured at different follow-up times might not be appropriate, to date, there is no consensus on the appropriate duration of a remineralising treatment cycle. In order to synthesise the data as much as possible, we will divide the time points into short-term (< 3 months) and long-term (> 3 months) and will extract the outcome data from the longest follow-up (closest to 3 months or longest after 3 months, respectively).

We will conduct both fixed-effects and random-effects model NMA to synthesise all evidence for each outcome. The choice between models will be based on the expectation of whether the intervention effects are truly identical or the funnel plot asymmetry or other conditions [43]. We will obtain a comprehensive ranking of all treatments (ranking probabilities, the surface under the cumulative ranking curve (SUCRA), and mean ranks) [32]. The estimated relative ranking of interventions will be generated according to primary outcomes. If the NMA and SUCRA cannot be performed on primary outcomes, we will refer to the NMA and SUCRA results of secondary outcomes.

### Geometry and feasibility of the network

We will explicitly describe the process leading to node grouping [44, 45]. The network of treatments will be judged based on the characteristics of the available studies and presented and evaluated graphically. We will evaluate the following: (1) if the network is disconnected, (2) if there is a sufficient number of comparisons in the network with available direct data, (3) if there is a high number of comparisons based on a single study, and (4) if any key treatments are missing. Next, the feasibility of the network meta-analysis will be assessed checking the following: (1) transitivity (i.e., the comparable distribution of effect modifiers across comparisons), which will be examined using boxplots or percentages to visually inspect potential effect modifiers of treatment effect [46]; (2) consistency between direct and indirect estimates of the effects, which will be examined using the node-splitting method [47], and globally (i.e., evaluating the network as a whole) using the design-by-treatment interaction model [48]; and (3) the amount of variability, which we will quantify, that can be attributed to heterogeneity and inconsistency rather than sampling error, by calculating the *I*^2^ statistic [49].

We will include both two-arm trials and multi-arm trials, and those comparing different active ingredients or dosages of the same active ingredients will be analysed separately for a global analysis of all outcomes. Different concentrations of the same active ingredient at the same dosage will be merged together for a global analysis of all outcomes (e.g., 900 ppm NaF toothpaste and 1100 ppm NaF toothpaste), with the exception of huge concentration differences (e.g., 900 ppm NaF toothpaste and 5000 ppm NaF toothpaste). Moreover, we will not distinguish interventions according to the duration and administration frequencies. Both the placebo and treatment-free groups will be merged together as the control group for global analysis of all outcomes.

### Risk of bias in individual studies

Two review authors will independently assess the risk of bias for each study using the criteria outlined in section 8.2 of the Cochrane Handbook for Systematic Reviews of Interventions (Higgins 2019) and in section 3.5 of Chaimani’s study (2017) [34, 50]. All disagreements will be resolved by discussion or by involving a third assessor. We will assess the following domains as ‘low’, ‘unclear’, or ‘high’ risk of bias:

- Sequence generation (selection bias)
- Allocation concealment (selection bias)
- Blinding of participants and personnel (performance bias)
- Blinding of outcome assessment (detection bias)
- Incomplete outcome data (attrition bias)
- Selective outcome reporting (reporting bias)
- Other bias

We will report these assessments in a ‘Risk of bias’ table for each included study and we will provide supporting judgments for each assessment.

We will provide summary assessments of the risk of bias for each important outcome (across domains) within and across studies following Table 8.7.a in the Cochrane Handbook for Systematic Reviews of Interventions (Higgins 2011) [50]:

- Low risk of bias (plausible bias unlikely to seriously alter the results) if all domains were assessed as at low risk of bias
- Unclear risk of bias (a plausible bias that raises some doubt about the results) if one or more domains were assessed as at unclear risk of bias
- High risk of bias (a plausible bias that seriously weakens confidence in the results) if one or more domains were assessed as at high risk of bias

### Statistical analysis

We will perform our NMA model with contrast-level data by multivariate meta-analysis (commands network meta and mvmeta) in STATA (version 14, Stata Corp, College Station, TX, USA) within a frequentist framework. The restricted maximum likelihood method will be used to estimate the between-study variance in the NMA [51, 52]. We will perform both traditional pairwise analyses and NMA. If NMA is not appropriate due to high global inconsistency or other conditions, a pairwise meta-analysis only will be considered. If a pairwise meta-analysis is also not possible, studies will be summarised narratively.

The outcomes of continuous and dichotomous variables will be presented as MDs and RRs with 95% CIs.

A network plot will be created to show what treatments can be compared as many as possible (Fig. 1). The effectiveness of each treatment among all treatments will be ranked, and we will generate plots of the treatment rank probabilities to rank the various treatments for each outcome.

### Unit of analysis issues

The participants will be the unit of analysis. Where cluster-randomised trials are included, we will undertake data analysis at the same level as the randomisation or at the individual level accounting for the clustering. In doing so, we will follow the advice provided in section 23.1.3 of the Cochrane Handbook for Systematic Reviews of Interventions [53].

### Dealing with missing data

In studies where data are unclear or missing, we will contact the principal investigators. If missing data are unavailable, we will follow the advice given in Section 10.12.3 of the Cochrane Handbook for Systematic Reviews of Interventions [43]. If results are only reported graphically, we will graphically obtain the values, whenever applicable.

### Assessment of heterogeneity

Heterogeneity will be assessed based on the characteristics and design of the included studies. Major sources of clinical heterogeneity included age, sex, and race. Different study designs and risks of bias may contribute to methodological heterogeneity. If substantial heterogeneity is identified, we plan to explore and address heterogeneity in a subgroup analysis or meta-regression. We will use the chi-squared test to identify subgroup differences. We will consider a *p* value < 0.05 as statistically significant.

### Assessment of transitivity and similarity

We will assess the assumptions of transitivity and similarity based on clinical and methodological characteristics. We will assume that intervention effects are transitive in this network meta-analysis, and we will investigate similarity based on clinical characteristics, such as the same mechanism of treatment, the similar period of treatment, and the severity of WSLs at baseline. All of these effect modifiers will be judged and reported before the network meta-analysis is conducted.

### Assessment of inconsistency

We will assess evidence for consistency in three ways: loop, design-by-treatment interaction, and node/side-splitting [47, 48, 54–57]. First, when there is a loop connecting three or more treatments, it is possible to evaluate the consistency between direct and indirect evidence. Second, we will use the design-by-treatment interaction model that provides a single inference, using the chi-squared test, about the plausibility of assuming consistency throughout the entire network. Third, we will use the node-splitting method to calculate the inconsistency of the model, which separates evidence on a particular treatment contrast into direct and indirect evidence.

### Assessment of reporting biases

If there are 10 or more studies in a meta-analysis, we will investigate reporting biases (such as publication bias) using funnel plots. We will assess the asymmetry of funnel plot by visual evaluation and statistical tests. For continuous outcomes, we will use the test proposed by Egger (1997), and for dichotomous outcomes, we will use the test proposed by Harbord [58, 59]. If asymmetry is detected in any of these tests or is suggested by visual assessment, we will perform exploratory analyses to investigate it.

### Sensitivity analysis

We will carry out sensitivity analyses to assess the robustness of our review results. Sensitivity analysis will be conducted by repeating meta-analysis with studies with an unclear or low risk for bias. More issues suitable for sensitivity analysis will be identified during the review process. We will report sensitivity analyses by producing a summary table [43].

### Summarising findings

We will create a ‘Summary of findings’ table for the comparisons of the primary and secondary outcomes. We will also describe the use of additional summary measures assessed, such as treatment rankings and SUCRA values, as well as modified approaches used to present summary findings from meta-analyses.

### Assessing the quality of the evidence

We will use the GRADE system (GRADE 2004) and the GRADEpro GDT software 2015 to create the table [60]. We will consult the latest literature on GRADE for network meta-analyses quality evaluation [61–64]. Four steps will be used to assess the quality of treatment effect estimates from NMA: (a) present direct and indirect treatment estimates for each comparison of the evidence network, (b) rate the quality of each direct and indirect effect estimate, (c) present the NMA estimate for each comparison of the evidence network, and (d) rate the quality of each NMA effect estimate [61, 62].

### Difference between the protocol and the review

All differences between the protocol and the final review will be reported with the rationale for these changes. We will also report the influences of these modifications.

## Discussion

### Strengths and limitations

This protocol uses direct and indirect comparison methods, which helps in determining the best agents before and after the occurrence of WSLs. By integrating currently available clinical data, the network meta-analysis will provide a high-quality conclusion for patients and clinicians about the prevention and treatment of the WSLs. Potential limitations and challenges include but are not limited to study level (the possibility of clinical heterogeneity, poor-quality reporting in the included trials, bias like patient intervention self-administration without the study assessing compliance with the instructions, analyses disregarding the clustering of measurements) and review level (a lack of available treatment comparisons to build robust nodes, the network unable to cover all the interventions and combinations). Summary and implications including the conclusion of recommended or ineffectual interventions/combinations will be discussed in the final manuscript.

### Importance and beneficiaries

This network meta-analysis will compare the effectiveness of remineralising agents in the prevention and treatment of orthodontically induced WSLs. Previous systematic reviews have only done pairwise comparisons. One study made a preliminary attempt on network plotting, but it was still a traditional meta-analysis [28]. For a better understanding of the effectiveness of various conservative WSL treatments, we will conduct a network meta-analysis that allows us to estimate the relative effectiveness of all available treatments. Simultaneously, it will improve the efficiency in comparative effectiveness research and in the quality of decision-making. We believe that the results of this review will be beneficial for clinical decision-making and will also advance future clinical studies.

### Amendments

Any amendments (such as nodes combination, results reporting) made to the current protocol will be published as a supplement in the final manuscript, any amendments made to this protocol when conducting the study including the date of and rationale for the amendments, with the final manuscript.

## Supplementary information

**Additional file 1.** PRISMA-P checklist.

**Additional file 2.** MEDLINE search strategy.

**Additional file 3.** PRESS Guideline — Search Submission & Peer Review Assessment.

**Additional file 4.** Characteristics of included studies.

## Data Availability

All data generated and analysed during this study will be included in the published article.

## Abbreviations

CI: Confidence intervals
CPP-ACFP: Casein phosphopeptide-amorphous calcium phosphate with fluoride
CPP-ACP: Casein phosphopeptide-amorphous calcium phosphate
HAP: Hydroxyapatite
MD: Mean difference
NMA: Network meta-analysis
PRISMA-P: The Preferred Reporting Items for Systematic Review and Meta-analysis Protocols
RR: Relative risk
SUCRA: Surface under the cumulative ranking curve
WSLs: White spot lesions
