# Supplementary material for: Effectiveness of remineralising agents in prevention and treatment of orthodontically induced white spot lesions: a protocol for a systematic review incorporating network meta-analysis
Source: Syst Rev. 2019 Dec 29;8:339. doi: 10.1186/s13643-019-1253-8 (PMC6935494; doi:10.1186/s13643-019-1253-8)
Supplement: Supplementary file 2 — Additional file 2. MEDLINE search strategy. [file 13643_2019_1253_MOESM2_ESM.docx]

Additional file 2

Medline(via Ovid) search strategy

1. exp Orthodontics/ 54310
2. (orthodontic*).mp. 83009
3. or/1,2 92363
4. exp Dental Caries/ 46532
5. (dental adj5 caries).mp. 68666
6. (Dental adj5 Deca*).mp. 5604
7. (Carious adj5 Dentin*).mp. 2216
8. (White adj5 Spot*).mp. 12864
9. Exp Decalcification, Pathologic/ 290
10. decalcificat*.mp. 9977
11. exp Tooth Demineralization/ 48154
12. (T??th adj5 Deminerali*).mp. 4589
13. (T??th adj5 Hypominerali*).mp. 416
14. (T??th adj5 cari*).mp. 20687
15. (Incipient adj5 caries lesion*).mp. 202
16. (Early adj5 caries lesion*).mp. 459
17. (enamel adj5 def*).mp. 4618
18. (enamel adj5 lesion?).mp. 4174
19. (enamel adj5 deca*).mp. 1004
20. (enamel adj5 cari*).mp. 5453
21. or/4-20 107546
22. exp Tooth Remineralization/ 2141
23. (t??th adj5 reminerali*).mp. 2945
24. CPP-ACP.mp. 758
25. CPP-ACFP.mp. 71
26. exp Fluoride/ 38591
27. fluorid*.mp. 143983
28. casein phosphopeptide amorphous calcium fluoride phosphate.mp. 44
29. casein phosphopeptide amorphous calcium phosphate.mp. 782
30. bioactive glass.mp. 3580
31. (prevent* adj5 cari*).mp. 16763
32. (prevent* adj5 white spot**$**).mp. 264
33. or/22-32 161048
34. exp Randomized Controlled Trial/ 488755
35. Randomized Controlled Trial.mp. 1114107
36. or/34,35 1116510
37. and/3,21,33,36 274
