# Supplementary material for: Effectiveness of remineralising agents in prevention and treatment of orthodontically induced white spot lesions: a protocol for a systematic review incorporating network meta-analysis
Source: Syst Rev. 2019 Dec 29;8:339. doi: 10.1186/s13643-019-1253-8 (PMC6935494; doi:10.1186/s13643-019-1253-8)
Supplement: Supplementary file 4 — Additional file 4. Characteristics of included studies. [file 13643_2019_1253_MOESM4_ESM.docx]

Study characteristics table

| author | year of publication | study design | number of arms | sample size | duration of follow-up | withdrawals | Randomization (individual or cluster） |
| --- | --- | --- | --- | --- | --- | --- | --- |
|  |  |  |  |  |  |  |  |

Patients characteristics table

| Study | Number of patients in each arm | age | gender |
| --- | --- | --- | --- |
|  |  |  |  |

Intervention and comparator details

| Study | Intervention classification | intervention performer | materials and techniques used | active ingredients | concentration/dosage form | frequency and duration |
| --- | --- | --- | --- | --- | --- | --- |
|  |  |  |  |  |  |  |

Outcomes (primary and secondary outcomes, adverse events)

| Study | Arm 1 and number of patients | Arm 2 and number of patients | Arm 3 and number of patients | Arm 4 and number of patients | Outcome 1 | Time of follow-up | Outcome data | Outcome 2 | Time of follow-up | Outcome data |
| --- | --- | --- | --- | --- | --- | --- | --- | --- | --- | --- |
|  |  |  |  |  |  |  |  |  |  |  |
|  |  |  |  |  |  |  |  |  |  |  |

Notes

| Study | sponsorship/funding | conflicts of interest |
| --- | --- | --- |
|  |  |  |
