# Supplementary material for: Effectiveness of remineralising agents in prevention and treatment of orthodontically induced white spot lesions: a protocol for a systematic review incorporating network meta-analysis
Source: Syst Rev. 2019 Dec 29;8:339. doi: 10.1186/s13643-019-1253-8 (PMC6935494; doi:10.1186/s13643-019-1253-8)
Supplement: Supplementary file 3 — Additional file 3. PRESS Guideline — Search Submission & Peer Review Assessment. [file 13643_2019_1253_MOESM3_ESM.docx]

# Appendix A

***PRESS Guideline* — Search Submission & Peer Review Assessment**

**SEARCH SUBMISSION: THIS SECTION TO BE FILLED IN BY THE SEARCHER**

| Searcher:Chong Feng | Email:tjfengchong@163.com |  |
| --- | --- | --- |
| Date submitted:Oct 8^th^, 2019 | Date requested by:Oct 7^th^, 2019 | *[Maximum = 5 working daydays]* |

**Systematic Review Title:**

**Effectiveness of remineralising agents in prevention and treatment of orthodontically induced white spot lesions: a systematic review incorporating network meta-analysis**

This search strategy is …

| √ | My PRIMARY (core) database strategy — First time submitting a strategy for search question and database |
| --- | --- |
|  | My PRIMARY (core) strategy — Follow-up review NOT the first time submitting a strategy for search question and database. If this is a response to peer review, itemize the changes made to the review suggestions |
|  | SECONDARY search strategy— First time submitting a strategy for search question and database |
|  | SECONDARY search strategy — NOT the first time submitting a strategy for search question and database. If  this is a response to peer review, itemize the changes made to the review suggestions |

**Database** (i.e., MEDLINE,CINAHL…):	*[mandatory]*

1.Medline

2.Embase

3.Cochrane Library (Central)

4.Web of science

5.Scopus

6.CNKI

7.WanFang

8.VIP

9.CBM

**Interface**

(i.e., Ovid, EBSCO…):	*[mandatory]*

1.Ovid

2.Ovid

3.Cochrane Library

4.Web of knowledge

5.Scopus

6.CNKI

7.WanFang

8.VIP

9.SinoMed

**Research Question**

(Describe the purpose of the search)	*[mandatory]*

This study is aimed to compare the efficacy of current remineralizing agents in the prevention and treatment of orthodontic WSLs by Bayesian NMA on RCTs.

**PICO Format**

(Outline the PICOs for your question — i.e., Patient, Intervention, Comparison, Outcome, and Study Design — as applicable)

| **P** | Patients had orthodontic WSLs after debonding, or patients will receive full-mouth fixed orthodontic appliance irrespective of their age, sex, race, nationality and socio-economic status. |
| --- | --- |
| **I** | Remineralized agents, including fluoride materials, CPP-ACP(casein phosphopeptide-amorphous calcium phosphate), bioactive glass. We will distinguish different forms/active ingredients of remineralized materials as different interventions (e.g., NaF varnish and difluorosilane varnish will be distinguished as two interventions). We will include studies of all combinations and concentration of these agents. |
| **C** | Remineralized agents against each other, against placebo/no treatment, or combinations of remineralized agents, such as the combination of fluoride and CPP-ACP.Any other kind of remineralized agents. |
| **O** | For debonding patients, the primary outcomes are: regression or disappearance of WSLs measured by any quantitative methods, such as fluorescent light techniques or WSL indexes. For patients who will receive fixed orthodontic appliance, the primary outcome is the prevalence of WSLs, and secondary outcomes are also WSLs quantitative methods. |
| **S** | Only RCTs (randomized clinical trials) will be included. |

**Inclusion Criteria**

(List criteria such as age groups, study designs, etc., to be included) *[optional]*

| **P** | - Patients with at least one white spot lesion on the labial surface of the teeth induced by fixed orthodontic treatment - Patients who will receive fixed orthodontic treatment and will be observed about WSLs. - No restrictions on patients’ sex, age, city, country, ethnicity, and socio-economic status. |
| --- | --- |
| **I** | - Remineralized agents used for already formed WSLs induced by orthodontic treatment or prevention of orthodontically induced WSLs formation. - Different forms/active ingredients of remineralized materials will be distinguished as different interventions. |
| **C** | - No treatment or placebo.   Any other kind of remineralized agents. |
| **O** | - Lesion severity (measured by WSL index, enamel decalcification index, DIAGNOdent pen reading, quantitative light-induced fluorescence, etc). - Lesion transition (progression, stability or regression). - WSLs prevalence (in the prevention of WSLs). - Other outcomes evaluate WSLs. |
| **S** | - Randomized controlled trials (parallel or clustered) |

**Exclusion Criteria**

(List criteria such as study designs, date limits, etc., to be excluded) *[optional]*

| **P** | - [Laboratory](javascript:;) [animal](javascript:;) - Patients with WSLs but were not induced by orthodontic treatment. - Patients with any illness potentially affecting the study outcome, such as enamel hypoplasia, craniofacial deformities, ongoing medication and so on. |
| --- | --- |
| **I** | - Non-remineralized methods for prevention and treatment orthodontically induced WSLs, such as bleaching, micro-abrasion, resin infiltration, etc. |
| **C** | - |
| **O** | - |
| **S** | - Non-randomized prospective or retrospective studies - Split-mouth trials, which are susceptible to “carry-across effect” and the resultant bias. - Case reports/ case series - Non-clinical studies (in vitro, ex vivo, in silico, etc) - Systematic reviews |

**Was a search filter applied?**

Yes	No

**If YES, which one(s) (e.g., Cochrane RCT filter, PubMed Clinical Queries filter)? Provide the source if this is a published filter.** *[mandatory if YES to previous question* — *textbox]*

PubMed RCT filter and Embase RCT filter recommended by Harvard library.

Pubmed: "randomized controlled trial"[pt]

Embase: 'randomized controlled trial'/exp

Other notes or comments you feel would be useful for the peer reviewer? ***[optional]***

https://guides.library.harvard.edu/c.php?g=309982&p=2079544

McKibbon KA, Wilczynski NL, Haynes RB; Hedges Team. Retrieving randomized controlled trials from medline: a comparison of 38 published search filters. Health Info Libr J. 2009 Sep;26(3):187-202. PubMed PMID: [19712211](http://www.ncbi.nlm.nih.gov/pubmed/19712211?dopt=AbstractPlus).

Please copy and paste your search strategy here, exactly as run, including the number of hits per line. ***[mandatory]***

**(Add more space, as necessary.)**

**Table 1** Medline(via Ovid) search strategy

1. exp Orthodontics/	54310
2. (orthodontic*).mp.	83009
3. or/1,2	92363
4. exp Dental Caries/	46532
5. (dental adj5 caries).mp.	68666
6. (Dental adj5 Deca*).mp.	5604
7. (Carious adj5 Dentin*).mp.	2216
8. (White adj5 Spot*).mp.	12864
9. Exp Decalcification, Pathologic/	290
10. decalcificat*.mp.	9977
11. exp Tooth Demineralization/	48154
12. (T??th adj5 Deminerali*).mp.	4589
13. (T??th adj5 Hypominerali*).mp.	416
14. (T??th adj5 cari*).mp.	20687
15. (Incipient adj5 caries lesion*).mp.	202
16. (Early adj5 caries lesion*).mp.	459
17. (enamel adj5 def*).mp.	4618
18. (enamel adj5 lesion?).mp.	4174
19. (enamel adj5 deca*).mp.	1004
20. (enamel adj5 cari*).mp.	5453
21. or/4-20	107546
22. exp Tooth Remineralization/	2141
23. (t??th adj5 reminerali*).mp.	2945
24. CPP-ACP.mp.	758
25. CPP-ACFP.mp.	71
26. exp Fluoride/	38591
27. fluorid*.mp.	143983
28. casein phosphopeptide amorphous calcium fluoride phosphate.mp.	44
29. casein phosphopeptide amorphous calcium phosphate.mp.	782
30. bioactive glass.mp.	3580
31. (prevent* adj5 cari*).mp.	16763
32. (prevent* adj5 white spot**$**).mp.	264
33. or/22-32	161048
34. exp Randomized Controlled Trial/	488755
35. Randomized Controlled Trial.mp.	1114107
36. or/33,34	1116510

37. and/3,21,33,46	274	We searched Medline database via Ovid on the July 24^th^, 2019.

**Table 2** Embase (via Ovid) search strategy

1. ‘Orthodontics’/exp	35030
2. ‘orthodontic*’	79624
3. or/1,2	81471
4. ‘Dental Caries’/exp	53009
5. ‘dental’ NEAR/5 ‘caries’	56834
6. ‘Dental’ NEAR/5 ‘Deca*****’	1678
7. ‘Carious’ NEAR/5 ‘Dentin*’	1016
8. ‘White’ NEAR/5 ‘Spot**$**’	3684
9. ‘Decalcification’/exp	146
10. ‘decalcificat*’	2453
11. ‘Tooth disease’/exp	231202
12. ‘T??th’ NEAR/5 ‘Deminerali*’	797
13. ‘T??th’ NEAR/5 ‘Hypominerali*’	161
14. ‘T??th’ NEAR/5 ‘cari*’	10045
15. ‘incipient’ NEAR/5 ‘caries lesion**$**’	59
16. ‘early’ NEAR/5 ‘caries lesion**$**’	153
17. ‘enamel’ NEAR/3 ‘def*’	1597
18. ‘enamel’ NEAR/5 ‘lesion**$**’	1693
19. ‘enamel’ NEAR/5 ‘deca*’	1618
20. ‘enamel’ NEAR/5 ‘cari*’	4555
21. or/4-20	241086
22. ‘t??th’ NEAR/5 ‘reminerali*’	380
23. ‘CPP-ACP’	344
24. ‘CPP-ACFP’.	25
25. ‘casein phosphopeptide amorphous calcium fluoride phosphate’	15
26. ‘casein phosphopeptide amorphous calcium phosphate’	415
27. ‘Fluoride’/exp	33186
28. ‘fluorid*’	72758
29. ‘prevent*’ NEAR/5 ‘cari*’	18403
30. ‘prevent*’ NEAR/5 ‘white spot**$**’	111
31. ‘bioactive glass’	2362
32. or/22-31	85802
33. ‘randomized Controlled Trial’/exp	558681
34. ‘randomized controlled trial’	732316
35. or/33,34	732627
36. and/3,21,32,35	233

We searched Embase database via Ovid on the July 24^th^, 2019.

**Table 3** Central (via Cochrane Library) search strategy

1. MeSH descriptor:[Orthodontics]explode all trees	2477

2. orthodontic*	4150

3. or/1,2	4924

4. MeSH descriptor:[Dental Caries]explode all trees	2287

5. dental caries	5004

6. Dental Deca*	966

7. Carious Dentin*	538

8. White Spot*	570

9. MeSH descriptor:[Decalcification, Pathologic]explode all trees	4

10. decalcificat*	145

11. MeSH descriptor:[Tooth Demineralization]explode all trees	2497

12. Tooth Deminerali*	721

13. Tooth Hypominerali*	32

14. incipient caries lesion*	80

15. early caries lesion*	210

16. enamel def*	521

17. enamel lesion*	772

18. Tooth cari*	3869

19. or/4-18	7173

20. MeSH descriptor:[Tooth Remineralization]explode all trees	296

21. tooth reminerali*	477

22. CPP-ACP	137

23. CPP-ACFP	8

24. casein phosphopeptide amorphous calcium phosphate	125

25. casein phosphopeptide amorphous calcium fluoride phosphate	67

26. MeSH descriptor:[Fluorides]explode all trees	2421

27. fluorid*	4835

28. bioactive glass	150

29. prevent* cari*	5063

30. prevent* white spot*	329

31. bioactive glass	150

32. or/20-31	128744

33. Randomized Controlled Trial	906278

34. and/3,19,32,33	381

We searched Embase database via Ovid in the July 24^th^, 2019.

**Table 4** Web of science (via Web of knowledge) search strategy

TS= orthodontic*	32145

2. TS=Dental Caries	41722

3. TS= Dental Deca*	18514

4. TS=Carious dentin	2319

5. TS= White Spot*	13222

6. TS= decalcificat*	3427

7. TS= T??th Deminerali*	5024

8. TS= T??th Hypominerali*	528

9. TS=early AND TS= caries lesion$	1169

10. TS=incipient AND TS= caries lesion$	354

11. TS=enamel def*	6529

12. TS=enamel lesion$	5511

13. TS=enamel deca*	2189

14. TS=T??th cari*	26424

15. or/2-14	78786

16. TS=t??th reminerali*	2692

17. TS= CPP-ACP	339

18. TS= CPP-ACFP	23

19. TS=casein phosphopeptide amorphous calcium fluoride phosphate	246

20. TS=casein phosphopeptide amorphous calcium phosphate	373

21. TS=fluorid*	142056

22. TS=bioactive glass	7503

23. TS= prevent* cari*	26381

24. TS= prevent* white spot$	980

25. or/16-24	173305

26. TS=randomized Controlled Trial	503336

37. and/1,15, 25,26	85

We searched Web of Science database via Ovid on the July 24^th^, 2019.

**Table 5** Scopus (via Scopus) search strategy

1. TITLE-ABS-KEY (orthodontic*)	65868

2. TITLE-ABS-KEY ("Dental Caries")	58488

3. TITLE-ABS-KEY ("Dental Deca*")	926

4. TITLE-ABS-KEY ("Carious dentin")	498

5. TITLE-ABS-KEY ("White Spot*")	5664

6. TITLE-ABS-KEY (decalcificat*)	4186

7. TITLE-ABS-KEY ("Tooth Deminerali*")	2268

8. TITLE-ABS-KEY ("Tooth Hypominerali*")	15

9. TITLE-ABS-KEY (early) AND TITLE-ABS-KEY ("caries lesion")	469

10. TITLE-ABS-KEY (incipient) AND TITLE-ABS-KEY ("caries lesion")	137

11. TITLE-ABS-KEY ("enamel def*")	1077

12. TITLE-ABS-KEY ("enamel lesion")	754

13. TITLE-ABS-KEY ("enamel deca*")	128

14. TITLE-ABS-KEY ("Tooth cari*")	703

15. or/2-14	70113

16. TITLE-ABS-KEY ("tooth reminerali*")	1854

17. TITLE-ABS-KEY ( CPP-ACP)	423

18. TITLE-ABS-KEY ( CPP-ACFP)	28

19. TITLE-ABS-KEY ("casein phosphopeptide amorphous calcium fluoride phosphate")	17

20. TITLE-ABS-KEY ("casein phosphopeptide amorphous calcium phosphate")	495

21. TITLE-ABS-KEY (fluorid*)	158722

22. TITLE-ABS-KEY ("bioactive glass"）	5650

23. TITLE-ABS-KEY ("prevent*") AND TITLE-ABS-KEY("cari*")	26587

24. TITLE-ABS-KEY ("prevent*") AND TITLE-ABS-KEY("white spot")	509

25. or/16-29	185388

26. ALL ("randomized Controlled Trial")	1704455

27. and/1,15,25,26	222

We searched Scopus database via Ovid on the July 24^th^, 2019.

**Table 6** CNKI (via CNKI) search strategy

SU='正畸' and SU=('白垩斑' + '白斑' + '脱矿' + '龋' ) and SU=('再矿化' + '治疗' + '干预' +

'预防') AND FT=('随机')

351

We searched CNKI database via Ovid on the July 24^th^, 2019.

*CNKI’s professional retrieval was applied. But CNKI’s professional retrieval does not support combining search hits. SU, subject; FT, full-text.

**Table 7** WanFang (via WanFang) search strategy

ALL(”正畸”) and ALL(”白垩斑” + ”白斑”+ ”脱矿”+ ”龋” )

and ALL(”再矿化” + ”治疗” + ”干预” + ”预防”) AND ALL(”随机”)

380

We searched WanFang database via WanFang in the March 1^st^, 2019.

*WanFang’s professional retrieval was applied. But WanFang’s professional retrieval does not

support combining search hits. All, full-text.

**Table 8** VIP (via VIP) search strategy

U(” 正畸”) and U(”白垩斑” + ”白斑”+ ”脱矿”+ ”龋”)

and U(再矿化” + ”治疗” + ”干预” + ”预防”) AND U(”随机”)

345

We searched VIP database via VIP in the March 1^st^, 2019.

*VIP’s professional retrieval was applied. But VIP’s professional retrieval does not

support combining search hits. U, full-text.

**Table 9** CBM (via SinoMed) search strategy

1. 正畸学"/exp	14682

2. 正畸	17499

3.or/1,2	18902

4. "龋齿"/exp	14052

5.龋	16008

6.白垩斑	54

7.白斑	4485

8.脱矿	1353

9.釉质缺损	46

10.or/4-9	18287

11. "牙再矿化"/exp	381

12. 再矿化	579

13.or/11-12	579

14.randomized controlled study/exp	404848

15.random*	1329140

16.or/14,15	1329140

22.and/3,10,13,16	215

We searched CBM database via SinoMed on the July 24^th^, 2019.

**PEER REVIEW ASSESSMENT: THIS SECTION TO BE FILLED IN BY THE REVIEWER**

|  | Reviewer:Shu Yu | Email: shuyu@scu.edu.cn | Date completed: Oct 10, 2019 | | |
| --- | --- | --- | --- | --- | --- |
|  |  |  |  | | |
|  | **1. TRANSLATION** |  |  | | |
|  | | A -­‐No revisions | | √ |  |
|  | | B -­‐ Revision(s) suggested | |  |  |
|  | | C -­‐ Revision(s) required | |  |  |

If “B” or “C,” please provide an explanation or example:

**2. BOOLEAN AND PROXIMITY OPERATORS**

| A -­‐No revisions | √ |
| --- | --- |
| B -­‐ Revision(s) suggested |  |
| C -­‐ Revision(s) required |  |

If “B” or “C,” please provide an explanation or example:

**3. SUBJECT HEADINGS**

| A -­‐No revisions | √ |
| --- | --- |
| B -­‐ Revision(s) suggested |  |
| C -­‐ Revision(s) required |  |

If “B” or “C,” please provide an explanation or example:

**4. TEXT WORD SEARCHING**

| A -­‐No revisions | √ |
| --- | --- |
| B -­‐ Revision(s)suggested |  |
| C -­‐ Revision(s) required |  |

If “B” or “C,” please provide an explanation or example:

**5. SPELLING, SYNTAX, AND LINE NUMBERS**

| A -­‐No revisions | √ |
| --- | --- |
| B -­‐ Revision(s)suggested |  |
| C -­‐ Revision(s) required |  |

If “B” or “C,” please provide an explanation or example:

**6. LIMITS AND FILTERS**

| A -­‐No revisions | √ |
| --- | --- |
| B -­‐ Revision(s) suggested |  |
| C -­‐ Revision(s) required |  |

If “B” or “C,” please provide an explanation or example:

OVERALL EVALUATION (Note: If one or more “revision required” is noted above, the response below must be “revisions required”.)

| A -­‐No revisions | √ |
| --- | --- |
| B -­‐ Revision(s) suggested |  |
| C -­‐ Revision(s) required |  |

Additional comments:

The search strategy is sound.
